# Dynamical Transition in Dehydrated Proteins

**DOI:** 10.1021/acs.jpclett.3c03584

**Published:** 2024-03-25

**Authors:** Johanna Kölbel, Moritz L. Anuschek, Ivonne Stelzl, Supawan Santitewagun, Wolfgang Friess, J. Axel Zeitler

**Affiliations:** † Department of Chemical Engineering, University of Cambridge, Cambridge CB3 0AS, U.K.; ‡ Department of Pharmacy - Center for Drug Research, Pharmaceutical Technology and Biopharmaceutics, Ludwig-Maximilians Universität, Butenandtstrasse 5, 81377 Munich, Germany

## Abstract

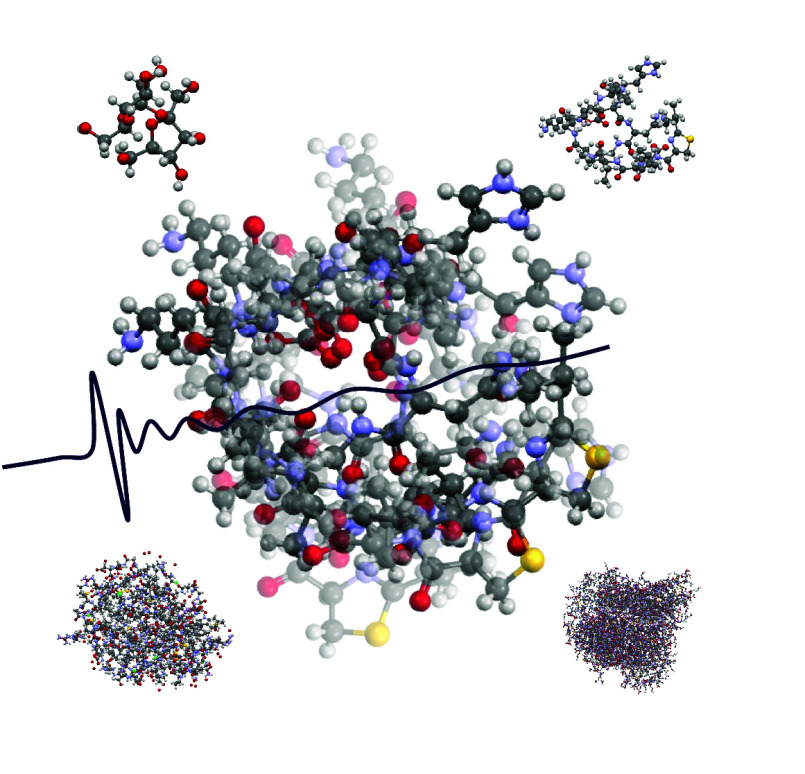

Terahertz time-domain spectroscopy and differential scanning calorimetry were used to study the role of the dynamics of biomolecules decoupled from solvent effects. Lyophilized sucrose exhibited steadily increasing absorption with temperature as anharmonic excitations commenced as the system emerged from a deep minimum of the potential energy landscape where harmonic vibrations dominate. The polypeptide bacitracin and two globular proteins, lysozyme and human serum albumin, showed a more complex temperature dependence. Further analysis focused on the spectral signature below and above the boson peak. We found evidence of the onset of anharmonic motions that are characteristic for partial unfolding and molecular jamming in the dry biomolecules. The activation of modes of the protein molecules at temperatures comparable to the protein dynamical transition temperature was observed in the absence of hydration. No evidence of Fröhlich coherence, postulated to facilitate biological function, was found in our experiments.

The number of protein drugs in therapy is steadily increasing. These biopharmaceuticals typically need to be administered by injection. Due to the intrinsically limited stability of the molecules in aqueous solution, the protein–drug products are frequently freeze-dried into an amorphous solid matrix for storage and reconstituted immediately before use.

The molecules surrounding the protein molecules in solution constitute their solvation shell (sometimes also called the hydration shell), and the molecular mobility of this shell affects the rates of conformational change, catalysis, and protein/DNA–protein interactions.^1,2^ The misfolding propensity and the pathways of protein aggregation depend on the protein’s local environment, which is influenced by the solvent, water, and sugars, salts, metal ions, and lipids that are part of the physiological environment or the reconstituted formulation. Molecular crowding at high protein concentrations, pH values, and buffer also plays a role.^3−8^ The predominant intermolecular interactions that proteins form with their surrounding environment are hydrogen bonds with water.

A recent paper^9^ highlighted the importance that the mobility of water plays in protein dynamics and, ultimately, aggregation. Simulations in combination with various experimental techniques focusing on the intrinsically disordered model protein α-synuclein (aSyn) showed that water mobility and aSyn mobility are inextricably linked. Enhancing the water mobility reduces the propensity of aSyn to aggregate.

The time scales of solvent motions and conformational changes in proteins differ significantly. Solvent motions are rapid, occurring on the femtosecond to picosecond time scale, whereas conformational changes in proteins happen on the nanosecond to millisecond time scale. Still, solvent mobility affects protein motions.^10^ The coupling of water motions, the presence of ions, and the protein dynamics are specific to the protein due to the differing charge distribution, hydrophobicity, and surface roughness.^11^

Thus, protein unfolding in solution occurs via a complex pathway and the properties of the hydration shell are critically important.^12^ These interactions are restrained in lyophilized samples with strongly reduced water content. Conversely, complete unfolding in the solid state is usually not observed, as thermal decomposition occurs before sufficiently high temperatures for unfolding are reached.

Conti Nibali et al. analyzed molecular dynamics simulations of a model protein with a focus on its complex vibrational landscape. They found multiple interfering acoustic-like and low-frequency optic-like modes, which suggest that protein collective dynamics are very complex. They hypothesize that anharmonic interactions and therefore the interactions between modes play an important role in the thermal transport as well as in the regulation of functional dynamical mechanisms.^13^

Instead of studying solvated biomolecules, we implemented an extensive lyophilization process to remove as many water molecules as possible from protein samples. We can thus study protein dynamics decoupled from solvent effects. This is of fundamental interest, as well as of practical importance, for lyophilisates of protein drugs.^14^

Lyophilisates typically comprise cryo- and lyoprotectants, surfactants, and the active biomolecule. The design of the products and the lyophilization process require a good understanding of the underlying stabilization mechanisms of the formulation components.^14,15^

In this context, the residual water content in the dried protein samples plays an essential role at low temperatures. Lyophilized lysozyme samples with residual hydration of >27% (m/m) water content, well above the typical water content of lyophilisates of ∼1% (w/w), exhibit the so-called protein dynamical transition (PDT).^16^ The PDT refers to an increase in the mean square displacement of molecules at a temperature of 180–220 K upon heating.^17−19^ The PDT, which is frequently measured with neutron scattering, is not to be confused with glass transition *T*_g_ that occurs in amorphous samples, which is characterized by a sudden change in relaxation times and heat capacity at *T*_g_ and is usually measured with DSC.^20^ The PDT has been observed at temperatures at ∼200 K for different lightly hydrated proteins and is thought to be due to the onset of motions involving interactions of charged side chains with surrounding water molecules.^21,22^

The PDT and the glass transition can both be described in terms of Goldstein’s potential energy landscape (or surface, PES) containing many small minima within larger basins.^23,24^ Moving between minima requires overcoming the local shallow activation energy barriers. At lower temperatures, the configurational entropy decreases; i.e., the number of available minima decreases. Moving from one basin to the next requires a large amount of activation energy and a cooperative rearrangement of molecules. The protein dynamical and glass transitions can be understood as corresponding to lowering energy barriers on that landscape. In the absence of hydration, a temperature increase activates a different set of motions of activation energies, similar to the thermal energies supplied. This effect was very subtle.

Given the propensity of hydrogen and van der Waals bonding interactions in solvated and lyophilized biopharmaceuticals, an ideal experimental method for studying such systems is terahertz time-domain spectroscopy (THz-TDS) due to the match in photon and bonding energies.^25^ In the terahertz range, the boson peak (BP) and the coupling of dipoles to the vibrational density of states dominate the absorption mechanisms.^26^ The motions at terahertz frequencies play an essential role in understanding solid-state protein dynamics. At storage temperature (room temperature), a considerable number of vibrational modes in the terahertz frequency range are active and contribute to formulation stability.

In the 1960s, Fröhlich suggested the existence of coherent vibrations at ∼10^11^ Hz^27−29^ and postulated that such motions enable biological functions, so-called biological control through selective long-range interactions. Motions active at terahertz frequencies are long-range motions, and the possible existence of a so-called Fröhlich condensate in lyophilized protein formulations can hence be investigated. Thus far, no experimental evidence for such coherent states was found for biomolecules in solution.

Vibrational confinement can dramatically reduce molecular mobility in lyophilisates at temperatures close to room temperature and depends heavily on the interaction between protein and excipients in the formulation.^30^ With an increase in temperature, the molecular mobility in the sample increases due to the ability to access more of the local minimum in the PES until the free volume is taken up entirely, and the molecule becomes “jammed”. Any further increase in mobility and hence terahertz absorption is no longer possible until a higher energy barrier to another basin in the PES is overcome that is associated with additional degrees of freedom for molecular motions.

In the spectral region that can be accessed with most terahertz spectrometers, between 0.3 and 3.0 THz (corresponding to the range between 10 and 100 cm^–1^, respectively), frequency-dependent infrared absorption coefficient α(ω) is theoretically related to the reduced density of states *g*(ω) via .^26^ The boson peak (BP) refers to an excess in the density of states with respect to Debye’s *g*(ω) ∝ ω^2^ law, which can appear as a peak in the reduced density of states. The BP is a harmonic phenomenon due to inherent disorder that anharmonic effects can obscure.^31^ Utilizing THz-TDS, the onset temperature of molecular mobility, previously termed *T*_g,β_, was found to correlate with anharmonic effects in the model glass former glycerol. These anharmonic effects resulted in an apparent shift of the BP center frequency and could thus be separated from purely harmonic contributions.^26^

The BP occurs close to the Ioffe–Regel crossover frequency at which the mean-free path of transverse waves becomes equal to their wavelength, meaning that there is a crossover from wave-like to random matrix-like physics. Once global mobility sets in above the glass transition temperature, anharmonicity and mobility increase with temperature until a “critical” mobility is reached, completely obscuring the BP.

Markelz et al.^32^ observed that in an amorphous sample, an increase in absorption with temperature at a single frequency as measured with THz-TDS is due to anharmonic effects, even at very low temperatures. While no actual PES is perfectly harmonic, these effects are comparatively small at low temperatures; e.g., the BP is not yet obscured or its apparent center frequency affected. We will hence refer to the temperature region below T*, i.e., at temperatures at which the BP is unaffected by anharmonic effects, as the harmonic regime, and to the temperature region at which the BP is affected as the anharmonic regime. This is expected to conceptually also apply to protein samples.

In the work presented here, we investigated terahertz protein dynamics decoupled from solvent effects in four different one-component lyophilized products, namely, sucrose (a widely used bulking agent, molecular weight of 0.34 kDa), bacitracin (a polypeptide antibiotic, molecular weight of 1.4 kDa), lysozyme (a globular protein, molecular weight of 14.5 kDa), and human serum albumin (HSA, a globular protein and bulking agent, molecular weight of 66.5 kDa), which are shown in Figure 1a. A possible Fröhlich condensate in lyophilized protein formulations would become apparent by distinct spectral features emerging in the terahertz spectrum.

**Figure 1:**
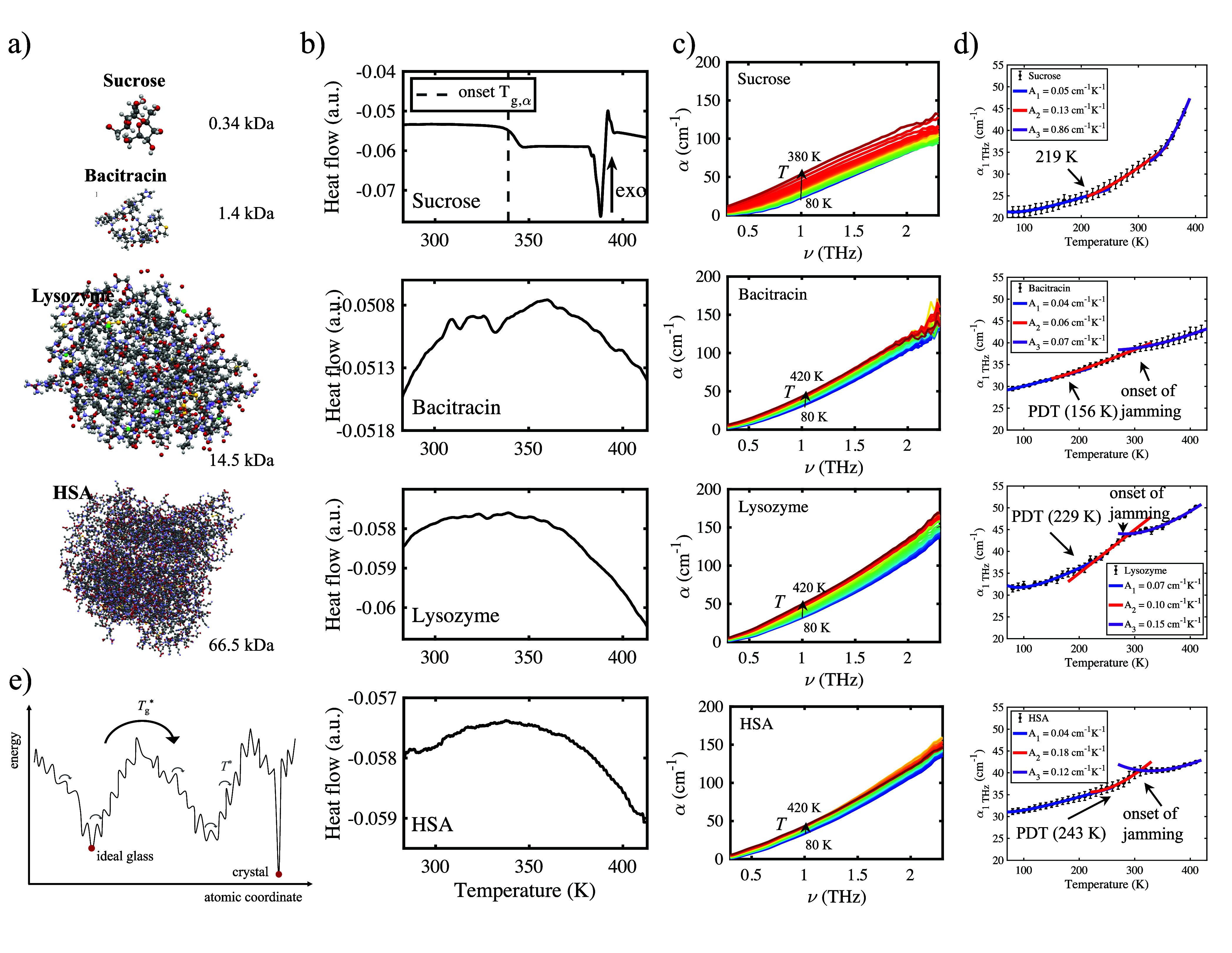
(a) Visualization of the four different key molecules of this study. (b) DSC results of sucrose, bacitracin, lysozyme, and HSA lyophilisates. A clear *T*_g_ was found for only sucrose. Inflection points for the other samples were found at 348 K (bacitracin), 330 K (lysozyme), and 337 K (HSA). (c) Terahertz spectra for sucrose, bacitracin, lysozyme, and HSA lyophilisates. Absorption mostly increases with temperature. Blue, 80 K; red, 420 K. (d) Absorption at 1 THz for sucrose, bacitracin, lysozyme, and HSA lyophilisates. Error bars are standard errors for *n* measurements (*n* = 5 for sucrose, *n* = 4 for bacitracin, and *n* = 3 for lysozyme and HSA). Fits are utilized to find transition temperatures (labeled with arrows), and fit coefficients *A*_1_–*A*_3_ are given in the legend. (e) Schematic of a possible potential energy landscape topology. With sufficient thermal energy, a sample can explore different hypersurface configurations and can become trapped in shallow minima.

In DSC data, a clear *T*_g_ was found for only sucrose [at 340 K (shown in Figure 1b)]. The peptide and proteins did not show a clear step in heat capacity, corresponding to *T*_g_; instead, a gradual decrease in heat flow was observed, potentially linked to structural changes at increased temperatures. The time scales of protein unfolding are strongly dependent on temperature, and it is possible that the mobility was insufficient to maintain equilibrium between folded and unfolded states during the DSC measurements.^33^ In all three pure macromolecule samples, an inflection point occurred, namely, at 348 K (bacitracin), 330 K (lysozyme), and 337 K (HSA).

The THz-TDS spectra of the four materials show a similar profile (Figure 1c). The change in the absorption coefficient with temperature was most pronounced for sucrose. Sucrose is an example of a model small molecular system that, in the disordered state, exhibits two glass transition temperatures (T* and *T*_g_^*^, previously termed *T*_g,β_ and *T*_g,α_, respectively^34,35^) upon heating from 80 K before crystallizing at ∼380 K.^36^Figure 1d shows the absorption coefficient extracted at a frequency of 1 THz for different samples. The absorption coefficient measures the change in dipole moments caused by inter- and intramolecular motions in the sample of interest. As the temperature increases, larger-scale motions become available, resulting in changes in the dipole moments. Each sample is characterized by a distribution of states, each with its own onset temperature. In smaller systems, such as sucrose, the distribution of states occurs over a narrower range of thermal energy, and the total number of states is lower than in more complex systems. Hence, in systems with a higher molecular weight, a glass or protein dynamic transition might not be well-defined by a single temperature. A broad distribution of dihedral angles corresponding to a number of shallow minima on the PES might instead lead to a more gradual transition. The observation of a sharp transition point instead might be an artifact of sparse temperature data.

This is indeed what we observe in the systems with a higher molecular weight. By utilizing a model with three fit parameters [α(1 THz, *T*) = *AT* + *B* + *C*/*T*], we can capture the temperature behavior of the samples well (Figure 1d). In the protein samples, there is considerable overlap among the fits in different temperature regimes, indicating a gradual transition. The model can be rationalized by considering the change in entropy with temperature and relating it to the absorption coefficient (details described in Materials and Methods). Glass or protein dynamical transitions change the fit parameters markedly, as is shown in Table 1 for linear parameter *A. A* is a measure for the rate of change in absorption with temperature at infinite temperatures, i.e., far from any phase transitions where the linear term dominates. Conversely, parameter *C* plays a larger role at lower temperatures. *C* can hence be related to the shape of a mainly harmonic potential, whereas *A* relates to a more anharmonic potential. While in sucrose, *A* increases >10-fold between the first and third temperature regime, the change is much smaller in the systems with a higher molecular weight. This again is an indicator that the PES of large lyophilized molecules comprises many shallow, largely harmonic minima, whereas anharmonicity plays a much larger role in sucrose, a system with fewer internal degrees of freedom. In sucrose, *A* increases at T* (219 K) and *T*_g_^*^ (343 K). *T*_g_^*^ agreed very well with the *T*_g_ measured by DSC (348 K) and literature values (between 325 and 349 K).^36^

**Table 1:** Linear Fit Parameters (10^–2^ cm^–1^ K^–1^) for Different Temperature Regimes in Sucrose, Bacitracin, Lysozyme, and HSA Lyophilisates

	sucrose	bacitracin	lysozyme	HSA
*A*_1_	4.6	3.7	7.4	3.9
*A*_2_	13.0	5.6	10.0	18.3
*A*_3_	86.4	7.2	15.2	12.1

It is known that the *T*_g_ of sucrose decreases with an increase in water content.^36^ The agreement between the *T*_g_^*^ values we measured with THz-TDS as well as DSC with literature values for dry sucrose matrices indicates that the experimental setup and procedures ensure a very low water content. Additionally in THz-TDS, any water molecules that could have potentially adsorbed to the sample surface during preparation may be removed by the vacuum (of <20 mbar) in the measurement chamber.

Generally, the rate of change in absorption with the temperature decreased at temperatures above 300 K for the higher-molecular weight systems. This phenomenon was previously observed in other (more complex) lyophilized formulations and attributed to high-temperature macromolecular confinement, strongly reducing the molecular mobility and resulting in a “jammed conformation”.^30^ We observed that the confinement effect depends on the shape and size of the molecules. This effect cannot be observed in small organic molecular systems like sucrose where only few degrees of freedom of dihedral motion are available, and hence, it is not possible to reach a “jammed conformation”. Bacitracin and lysozyme show a similar restriction of motions to that observed in HSA, i.e., a change of the PES. The effect is slightly more pronounced for lysozyme due to its increased size and hence higher number of internal degrees of freedom. Between 310 and 330 K, the absorption coefficient does not increase and the overall absorption change becomes smaller above 300 K. At temperatures above 330 K, the absorption increases again with temperature, as is also the case for BSA formulations measured previously.^30^ This temperature corresponds to an energy barrier of 2.7 kJ mol^–1^ (equal to 0.65 kcal mol^–1^, *k*_b_*T* at 330 K), which is significantly lower than the energy barrier of unfolding of BSA in solution, which is on the order of 64–267 kJ mol^–1^.^37^

This shows that structural changes in the solid state require only a small energy compared to complete unfolding in solution and can be attributed to the role of water surrounding the molecules in solution.^9^

The confinement is most pronounced in HSA, which is the largest macromolecule studied. Here, the absorption coefficient even decreases slightly at temperatures between 310 and 360 K. This decrease in absorption could be because the molecules may lose some of the degrees of freedom that they already gained at lower temperatures as the conformational jamming increases once they become trapped in a steep minimum on the potential energy landscape, as shown schematically in Figure 1e. Even after the jammed conformation is overcome, the change in absorption with temperature in HSA is much smaller compared to that in lysozyme.

The BP can be visualized by plotting absorption coefficient α divided by ν^3^ over frequency ν (Figure 2),^26^ and the maximum value of α/ν^3^ was found from smoothed data. If the BP occurs below 0.3 THz, the data are not extrapolated and no maximum is reported to avoid extrapolation errors.

**Figure 2:**
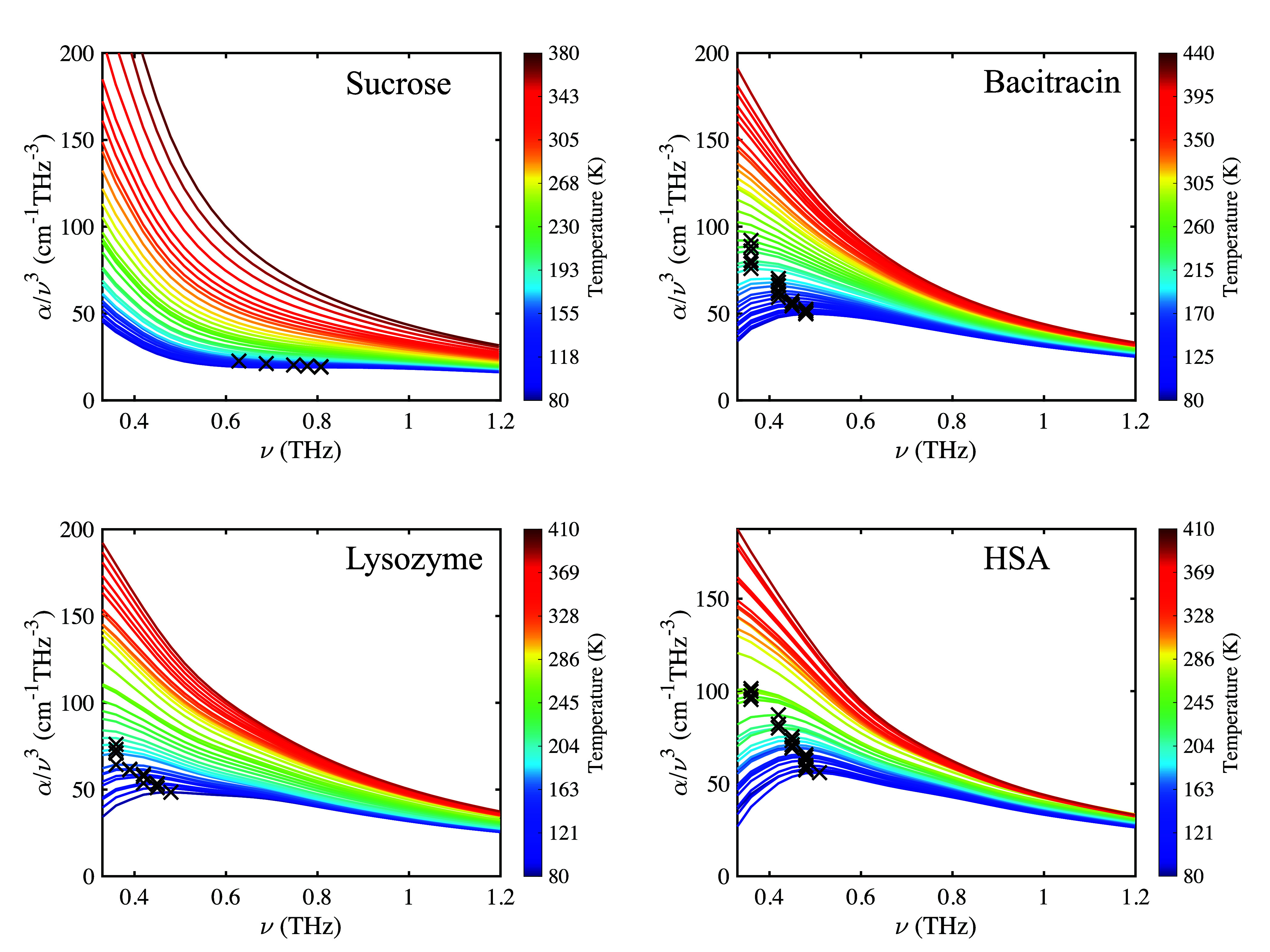
BP visualization for sucrose, bacitracin, lysozyme, and HSA lyophilisates at different temperatures. Boson peaks have been highlighted with crosses.

In small molecular systems, for example, in glycerol, the dynamics of glasses upon heating usually fall into two regimes.^38^ At very low temperatures, the terahertz spectra are dominated by harmonic excitations. The BP itself is harmonic and therefore a temperature-independent phenomenon. Its center frequency is constant. Above T*, the system leaves the harmonic minimum on the potential energy landscape. Shallower minima increase the amounts of anharmonicity and absorption. Once anharmonic effects dominate at T*, they lead to an apparent frequency shift of the BP maximum and obscure it completely at temperatures close to *T*_g_^*^.^26^

The maximum frequency of the BP of the protein lyophilisates is preserved in the harmonic regime at temperatures below ∼150 K. However, it decreases in the anharmonic regime with an increase in temperature before being obscured by anharmonic effects that appear to shift it outside the experimentally accessible region. The BP in sucrose is the least pronounced of the samples measured and appears to be masked by anharmonic effects at very low temperatures.

At very similar temperatures, the proteins reconfigure and become trapped in a different conformation, decreasing their mobility overall. Interestingly, the inflection point observed in the DSC data coincides with the temperature regime just above the initial trapping. Therefore, it is hypothesized that the trapping and/or the conformational change inducing the trapping results in a subtle heat capacity change with an increase in temperature and that the maximum in the DSC data corresponds to partial unfolding.

Derivative parameter *a* can be utilized to characterize terahertz spectra at frequencies above and below the BP more reliably. It reflects the slope of the absorption spectra averaged over a specific frequency range.^26^

We chose three different frequency ranges: 0.35–0.55 THz (below the BP), 0.90–1.10 THz (above the BP at the spectrometer’s highest signal-to-noise ratio), and 1.45–1.65 THz (at even higher frequencies above the BP).

Sucrose shows a pronounced increase in *a* over temperature, and a distinct increase is observed at *T*_g_^*^. This discontinuity is expected, as the glass transition temperature marks the onset of global mobility.

The lyophilisates show a markedly different behavior at frequencies below and above the BP (Figure 3a). In the protein samples, the plateau just above room temperature, seen in the absorption coefficient at 1 THz (Figure 1d), can be observed only at frequencies above the BP. At lower frequencies, the increase in *a* with temperature is monotonous. For bacitracin, that increase is approximately linear with temperature, while for lysozyme and HSA, subtle transitions can be observed at ∼200 and ∼300 K, respectively.

**Figure 3:**
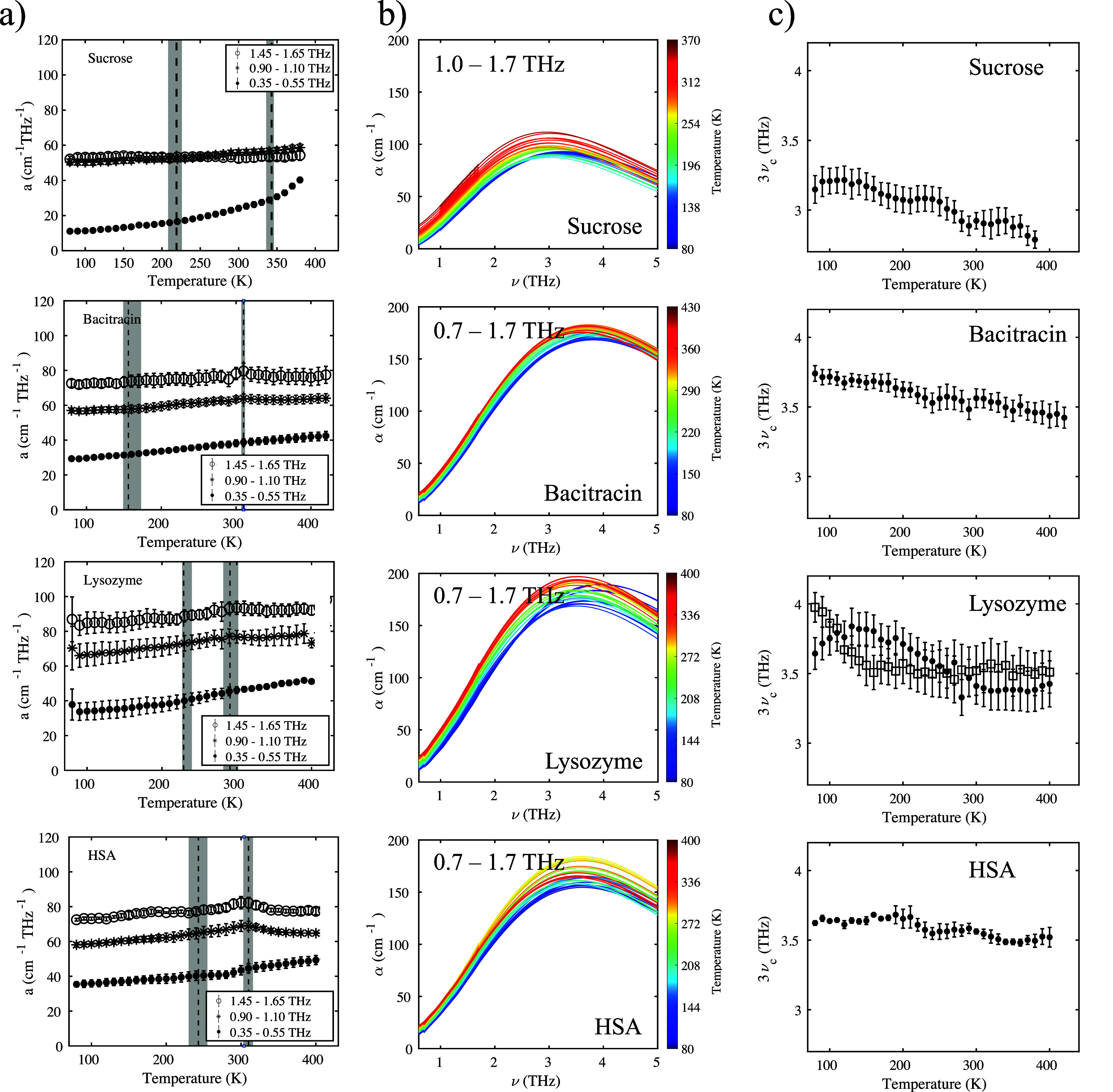
(a) Anharmonicity parameter evaluated in different frequency ranges for sucrose, bacitracin, lysozyme, and HSA lyophilisates. Error bars are standard errors for *n* measurements (*n* = 5 for sucrose, *n* = 4 for bacitracin, and *n* = 3 for lysozyme and HSA). Vertical lines denote transition temperatures found earlier, and shaded areas their associated uncertainties. (b) Extrapolated spectra at higher frequencies, for sucrose, bacitracin, lysozyme, and HSA lyophilisates, fit to frequencies above the BP maximum. (c) Extrapolated center frequency for sucrose, bacitracin, lysozyme, and HSA lyophilisates. Error bars are standard errors for *n* measurements (*n* = 5 for sucrose, *n* = 4 for bacitracin, and *n* = 3 for HSA). For the lysozyme lyophilizate, two separate measurements are shown with error bars showing the respective 95% confidence intervals of the fit.

Given that the increase in *a* appears in a temperature range similar to that of the PDT, for example, in lysozyme, it could be caused by side chain motions where the activation energy is not affected by the presence of the solvent.^32^

While the protein dynamical transition has thus far been observed only in hydrated samples, these increases in *a* point to the existence of thermally activated modes that involve only the protein molecules themselves. Even without solvent molecules, parts of the proteins retain some flexibility.

While the absorption coefficient at 1 THz is located relatively close to the BP maximum and is strongly influenced by anharmonic effects, a different behavior may be observed at higher frequencies.

In the following, we use a model developed by Chumakov et al.^38^ to investigate the effect of subtle spectral changes on the extrapolated vibrational density of states (VDOS). This model was previously utilized to investigate the model glass former glycerol and is now applied to more complex biomolecules.

The exponential function α = *A*ν^3^ exp(−ν/ν_c_) + *C* is fitted to the higher-frequency part of the experimentally accessible spectrum. If that function is plotted at even higher frequencies, a peak with a center frequency of 3ν_c_ is observed. It has to be emphasized here that this is a feature of the fitting function and not an accurate prediction of the center frequency of the VDOS. The available experimental data span a frequency range from the Ioffe–Regel crossover up to approximately 2.3 THz, while the actual VDOS may exhibit an underlying multipeak structure^39^ and show more features beyond the peak itself^40^ over a spectral range that cannot be accessed with the present spectrometer. The following discussion thus focuses mainly on how fit parameter ν_c_ depends on the temperature.

Chumakov et al. found excellent agreement in the frequency range of 1–1.7 THz in glycerol, where an exponential decay in the reduced density of states was observed.^38^ The reduced density of states and the absorption coefficient measured with THz-TDS are related by a factor of ν^3^.

While this model has been validated on the example of glycerol, it is based on the complex shear modulus in disordered solids. The only sample-specific parameter is phenomenological Raman coupling coefficient *C*(ω), which is *C*(ω) ∝ ω in most systems, including lysozyme,^41,42^ and the model can hence be applied to even these more complex systems.

The peak described by this model is the narrowest and least intense in sucrose and becomes broader but more intense in the protein samples (Figure 3b). Some temperature dependence of the center frequency is also apparent (Figure 3c). In all samples, the center frequency of the VDOS decreases with an increase in temperature (both in single measurements and in the averaged curves shown in Figure 3c), thereby shifting the VDOS to lower frequencies and increasing the absorption coefficient measured at the shoulder (e.g., at 1 THz). It is possible that the frequency shift may follow a Bose–Einstein distribution as previously observed for crystalline modes where thermal excitation was mediated by phonons populating an anharmonic potential. A red-shift of a mode is observed when phonons are excited by sufficient thermal energy.^43^ However, because the data are only extrapolated, we refrain from fitting a model and will simply discuss it in broader qualitative terms. In the future, it might be beneficial to measure similar samples on a spectrometer with higher spectral bandwidth to be able to extract more accurate data.

For sucrose, the change in the center frequency is pronounced above *T*_g_^*^, whereas in bacitracin, the decrease is gradual over the entire temperature range but slightly increased between 180 and 350 K. The center frequency of lysozyme generally decreases at temperature below 300 K and is constant above. The center frequency of HSA lyophilisates is constant upon heating to a temperature of 210 K, followed by a slight decrease in ν_c_. In all cases, the change in the center frequency is smaller at temperatures at which confinement is observed, indicating that the VDOS does not shift. If the molecules are trapped in a conformation, one may assume that the vibrations are independent of temperature.

The higher frequencies seem to be more affected by the activation of modes at ∼200 K. Modes at higher frequencies typically involve a reduced mass lower than that of low-frequency vibrations. The effect on the higher-frequency modes, therefore, is an indication that the modes that become active at ∼200 K may involve side chains or functional groups rather than the heavy protein backbone, which would influence the lower frequencies instead. In this respect, using a frequency of 1 THz to analyze the systems is beneficial as it provides insight into anharmonic effects, disappearance of the BP at low temperatures, and activation of modes as well as jamming at increased temperatures.

Fröhlich postulated the existence of coherent vibrations in the gigahertz to terahertz range that help facilitate the biological function of biomolecules.^29,44^ In terahertz spectra, such coherent modes appear as peaks in the absorption spectrum. Simultaneously, the absorption coefficient at neighboring frequencies decreases.^45^ No such features were found in any measurement (Figure 1c). Our data hence show no evidence for Fröhlich coherence or the existence of a Fröhlich condensate or other quantum effects in a system that is not actively being pumped.^46^ In protein solution, terahertz vibrations are propagated by the solvent and coupled to dielectric relaxations. This may lead to the fast dissipation of any postulated coherence or localized states due to the similarity in their frequencies. In this case, only extraordinarily rigid or well-ordered molecular structures would be observable. However, we do not find such evidence of inherent coherent states for the four molecules under investigation.

The importance of solvents and the solvation shell surrounding proteins for their function is widely recognized. In our work, we tried to obtain insights into protein–protein interactions in the dry state analyzing lyophilisates of sucrose, bacitracin, lysozyme, and HSA by terahertz spectroscopy.

The glass transition temperature, *T*_g_, was identified in sucrose by DSC, whereas *T*_g_ could not be identified for bacitracin, lysozyme, and HSA. However, THz-TDS demonstrated an increase in mobility with temperature. An increase in the temperature led to the activation of modes involving only the protein molecules, resulting in an increase in the absolute absorption coefficient and the anharmonicity parameter. The anharmonicity parameter showed a markedly different behavior below and above the BP center frequency. Utilizing the theoretical model by Chumakov et al., the higher frequencies were also evaluated, and a red-shift of the VDOS was predicted. Anharmonicity began to influence the spectra in sucrose at T* and resulted in an apparent shift of the center frequency of the BP. A further increase in temperature and, thereby, mobility led to the dissipation of the BP. For the larger proteins, lysozyme and HSA, jamming was observed at increased temperatures after the dissipation of the BP and could be overcome only by a further increase in temperature. Future experiments making use of a higher-bandwidth spectrometer can investigate the impact of the change in temperature on the VDOS. In the frequency range investigated, we could not find evidence for the occurrence of Fröhlich coherence.

Our experiments show that the protein dynamical transition can be observed for a wide range of biomolecules that are completely dry, supporting the findings reported by Liu et al. for two proteins and measured using neutron scattering techniques.^47^ The clear experimental observation of the dynamical transition (Figure 1d) in the absence of meaningful amounts of hydration water is in contrast to previous experimental and numerical data that assert the key role of water molecules in driving the dynamical transition behavior of the biomolecule.^22^ We comment that the dynamical transition might occur gradually over a range of temperatures and might therefore not necessarily be well-defined. Previous data were often collected with worse temperature resolution than in the study presented here, which might mask this behavior. It is important to emphasize that this does not contradict the critical role of the complex interplay in the dynamics between protein and bulk water in biological function,^9,48^ or the important role hydration water can play in the dynamical transition. However, the physical origin in the PDT is not the hydration water itself, but it is intrinsic to the protein. The onset of the dynamics, albeit fundamentally different from the specific concept outlined by Fröhlich, may well play an important role for the biological function of proteins in solution as well as for its structural stability in the solid state, such as in lyophilized products.

## Materials and Methods

Sucrose (a commonly used cryo- and lyoprotectant, molecular weight of 0.34 kDa) and HSA (globular model protein, molecular weight of 66.5 kDa) were purchased from Merck GmbH (Steinheim, Germany). Bacitracin (a polypeptide antibiotic, molecular weight of 1.4 kDa) and lysozyme (a globular protein, molecular weight of 14.5 kDa) were purchased from Carl Roth GmbH (Karlsruhe, Germany). The samples were prepared with highly purified water (HPW) (Sartorius Arium Pro, Sartorius, Göttingen, Germany) to reach a total solid content of 10% (m/m) prior to lyophilization.

*Lyophilization*. Lyophilization stoppers (B2-TR coating, West) and DIN 10R vials (Fiolax, Schott, Germany) were cleaned with highly purified water and dried at 333 K for 8 h. The vials were filled with 3 mL of a solution and subsequently semistoppered. The product temperature in vials at different positions on the shelf was recorded with a thermocouple. Formulations were freeze-dried according to the protocol in Table 2 using an FTS LyoStar 3 freeze-dryer (SP Scientific, Warminster, PA). The end of primary drying was controlled by comparative pressure measurement between a Pirani and an MKS sensor. The vials were stoppered after secondary drying under a nitrogen atmosphere at 800 mbar and crimped with flip-off seals. Karl Fischer headspace analysis confirmed that the moisture content of all samples was ≤0.2 wt %.

**Table 2:** Lyophilization Protocol

step	ramp (K/min)	shelf temperature (K)	pressure (μbar)	hold time (h)
freezing	1.0	223	1 atm	3
primary drying	0.5	253	60	50
secondary drying	0.4	323	60	5

*Differential Scanning Calorimetry (DSC)*. The *T*_g_ of the lyophilisates was determined with a DSC 821^e^ instrument (Mettler Toledo, Giessen, Germany). Then, 5–10 mg of crushed lyophilized cake was placed in 40 μL aluminum crucibles (Mettler Toledo) under controlled humidity conditions (≤10% relative humidity) and sealed hermetically. The samples were heated from 280 to 415 K at a ramp rate of 2 K min^–1^. The *T*_g_ was determined as the midpoint of the phase transition.

*Terahertz Time-Domain Spectroscopy (THz-TDS)*. The lyophilized cake was broken up under a dry nitrogen atmosphere contained within a glovebag (AtmosBag, Merck UK, Gillingham, U.K.), and the powder was gently mixed using an agate mortar and pestle and then pressed into a thin pellet (thickness of <800 μm, diameter of 13 mm) using a manual press (load 3 t, Specac Ltd., Orpington, U.K.). The pellet was sealed between two z-cut quartz windows with a thickness of 2 mm each and fixed to the coldfinger of a cryostat (ST-100, Janis, Wilmington, MA).

Samples were analyzed with a Terapulse 4000 spectrometer (Teraview Ltd., Cambridge, U.K.) in transmission under vacuum (pressure of <20 mbar). The reference spectrum of each sample was calculated from the coaverage of 1000 waveforms that were acquired with a resolution of 0.94 cm^–1^ and transformed into the frequency domain via fast Fourier transform. The absorption coefficient was calculated following the method by Duvillaret et al.^49^

At the beginning of each measurement, the sample was cooled from room temperature to 80 K and left to equilibrate for at least 30 min. The temperature was subsequently increased in steps of 10 K up to a maximum temperature of 440 K. The system was allowed to equilibrate for 8 min at each temperature increment before reference (two z-cut quartz windows with no sample between them) and sample measurement.

*Determination of Transition Temperatures*. Transition temperatures have previously been determined utilizing linear fits to the absorption coefficient extracted at 1 THz^25^ for different temperatures *T*. Here, we expand this method by including an additional 1/*T* term in the fit. This is motivated by considering entropy, *S*, which is assumed to change linearly with temperature outside of phase transition regions, as well as a partition-like function, , where *g*(*E*) is the density of states, *k* is the Boltzmann constant, and *E* is the energy. As recently shown,^26^ the density of states can in turn be related to the terahertz absorption coefficient. *S* and *Z* are related via the equation . At a single frequency, the absorption can hence be written as . The additional 1/*T* fit term has a greater impact on the fit at low temperatures. In sucrose, the additional term accounts for only 8% of the absorption at 200 K compared to 19% at 100 K. The physical meaning of the fit parameters is discussed above.

To determine the transition temperatures, fits to α(1 THz, *T*) in the temperature interval (*T*_1_, *T*_4_) are performed: , , and . *T*_2_ and *T*_3_ are varied in 10 K steps, and the root-mean-square error of the three respective fits is recorded. The *T*_2_ and *T*_3_ resulting in the lowest overall error are deemed the best, and the transition temperatures are calculated as the intersection of the two corresponding fits:  and , respectively.

It is known that sucrose exhibits three different temperature regimes, which are well reproduced with this fitting regime.^36,50^ The absorption coefficient of the more complex lyophilisates was also best described with three fit functions while avoiding overfitting. The increase in absorption most visible at ∼290 K in these samples could not be captured with the same fit that described the lowest temperatures.

Errors can be estimated after the best fit has been found by varying the temperature intervals around the optimum temperatures by ±10 K and calculating alternative transition temperatures. This results in nine pairs of transition temperatures. For a conservative estimate, the minimum and maximum of those values were chosen to represent errors, while using their standard deviation would also be possible.

## Supporting Information Available

Absorption at 1 THz for lyophilisates (Figure S1) and extrapolated maximum absorption for lyophilisates (Figure S2) (PDF)
Transparent Peer Review report available (PDF)
