# Supplementary material for: Dynamical Transition in Dehydrated Proteins
Source: J Phys Chem Lett. 2024 Mar 25;15(13):3581–90. doi: 10.1021/acs.jpclett.3c03584 (PMC11000241; doi:10.1021/acs.jpclett.3c03584)
Supplement: Supplementary file 1 — jz3c03584_si_001.pdf [file jz3c03584_si_001.pdf]

Supplemental materials to manuscript 'The Dynamical Transition in Dehydrated Proteins'

Johanna Kölbel,[†,§] Moritz L. Anuschek,[†,‡,‖] Ivonne Stelzl,[‡,¶] Supawan Santitewagun,[†] Wolfgang Friess,[‡] and J. Axel Zeitler[*,†]

†*Department of Chemical Engineering, University of Cambridge, Cambridge, CB3 0AS, UK*

‡*Department of Pharmacy – Center for Drug Research, Pharmaceutical Technology and Biopharmaceutics, Butenandtstrasse 5, 81377 Munich, Germany*

¶*Current affiliation: Coriolis Pharma Research GmbH, Fraunhoferstraße 18b, 82152 Martinsried, Germany*

§*Current affiliation: School of Engineering, Brown University, 184 Hope Street, Providence, RI 02912, USA*

‖*Current affiliation: Novo Nordisk, Novo Nordisk Park 1, 2760 Måløv, Denmark*

E-mail: jaz22@cam.ac.uk

Phone: +44 (0) 1223 334783. Fax: +44 (0) 1223 334796

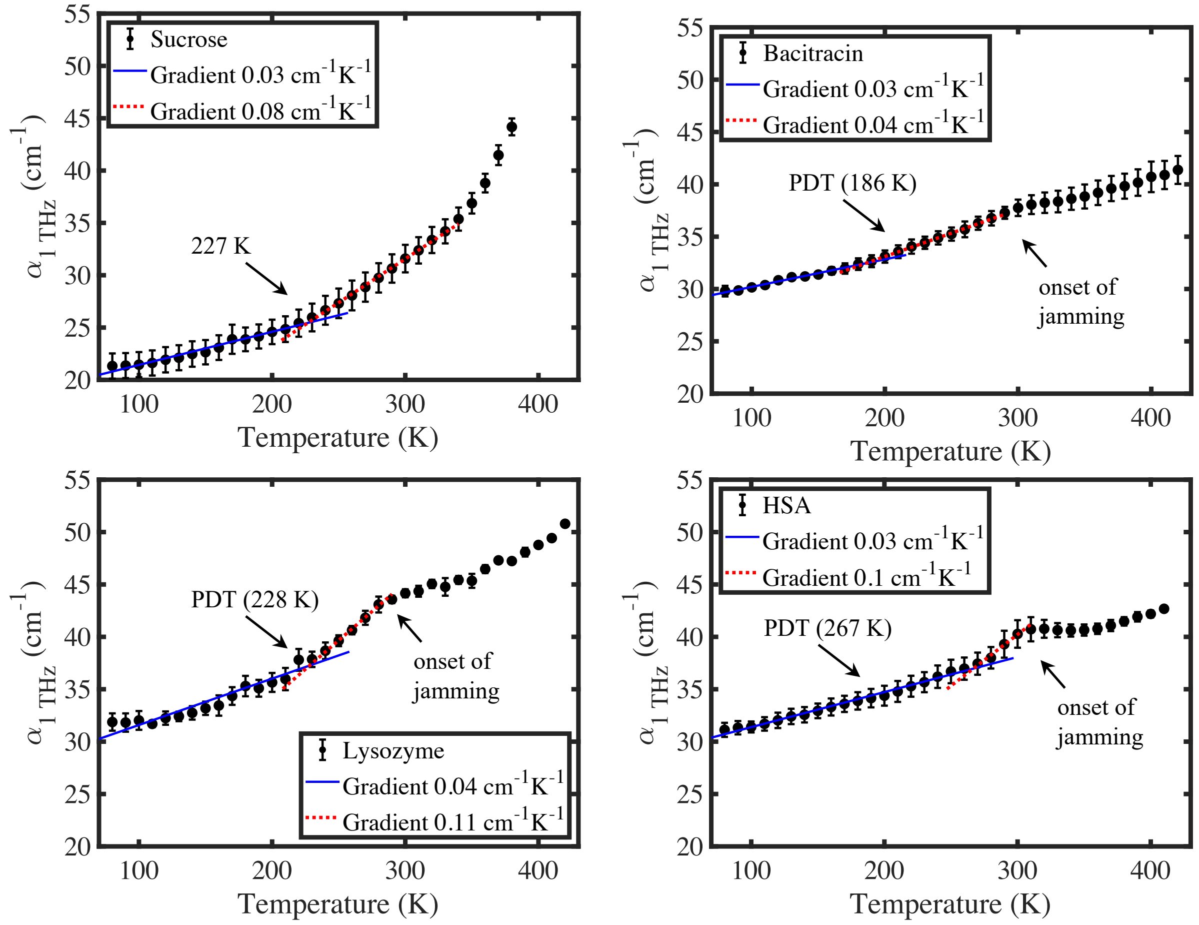

Figure 1: Absorption at 1 THz for sucrose, bacitracin, lysozyme, and HSA lyophilisates. Error bars are standard error for n measurements ($n = 5$ for sucrose, $n = 4$ for bacitracin, $n = 3$ for HSA). Linear fits are utilised to determine transition temperatures (labelled with arrows) and gradients are given in the legend.

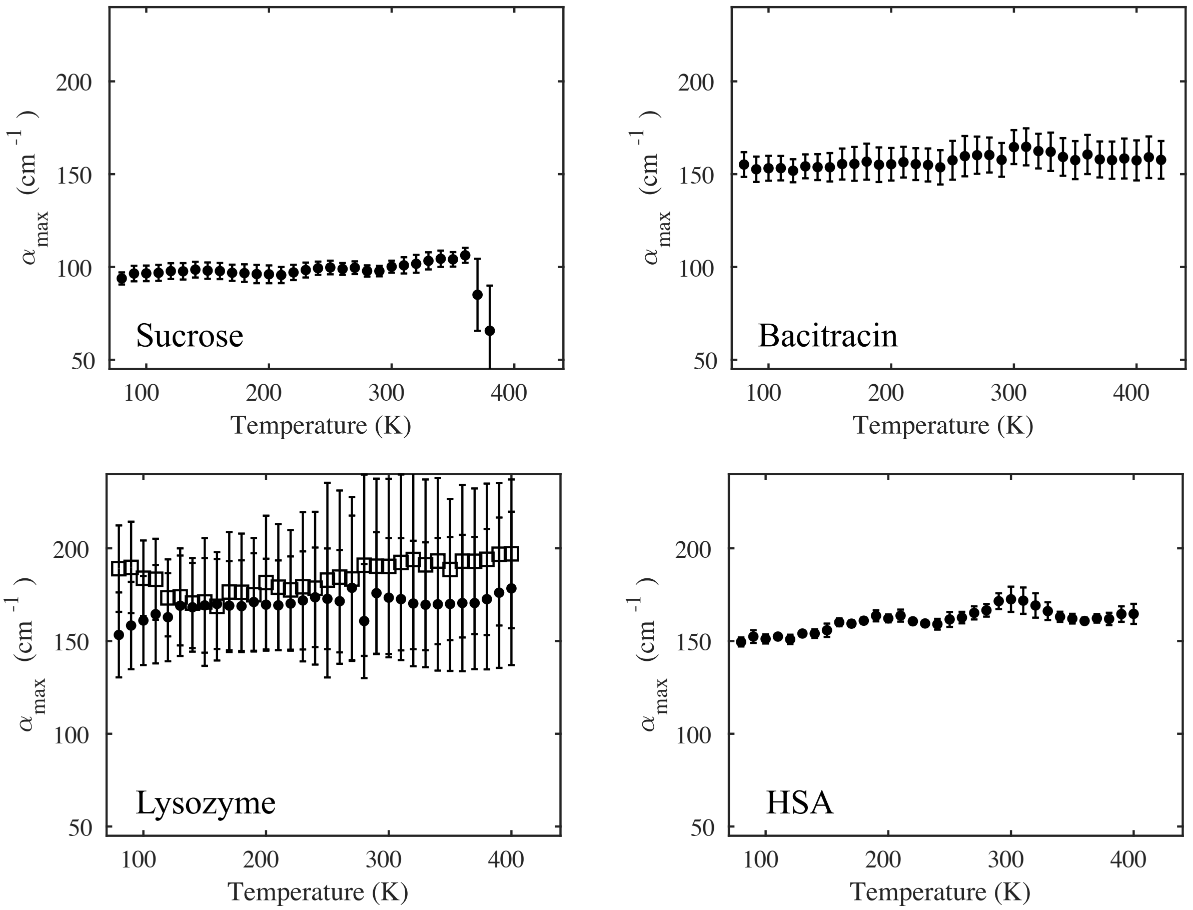

Figure 2: Extrapolated maximum absorption for sucrose, bacitracin, lysozyme, and HSA lyophilisates. Error bars are standard error for n measurements ($n = 5$ for sucrose, $n = 4$ for bacitracin, $n = 3$ for HSA). For the lysozyme lyophilisate, two separate measurements are shown with error bars calculated from the respective 95 % confidence intervals of the fit.
