# Supplementary material for: Dynamical Transition in Dehydrated Proteins
Source: J Phys Chem Lett. 2024 Mar 25;15(13):3581–90. doi: 10.1021/acs.jpclett.3c03584 (PMC11000241; doi:10.1021/acs.jpclett.3c03584)
Supplement: Supplementary file 2 — jz3c03584_si_002.pdf [file jz3c03584_si_002.pdf]

jz-2023-03584s.R1

Name: Peer Review Information for "The Dynamical Transition in Dehydrated Proteins"

First Round of Reviewer Comments

Reviewer: 1

Comments to the Author

I enjoyed reading this paper, which is suitable posed for JPC, and learnt a lot. So I recommend publication. Some minor comments:

The language of this paper is often unfamiliar with phys chem teaching and practice. With this comes new phenomena that physical chemists have not focused on before. Since the 19th century such spectra have been recorded in wavenumbers not THz. The use of such a physically meaningful unit provides insight that can never be provided when SI units are used. This paper already uses wavenumbers whenever physical insight is evoked from the readers, but the main results are not presented in widely understood language. If I have done it right, then the frequency range used in the paper of 0.3 to 3 THz corresponds to 10-100 wavenumbers … telling people this tells the audience what it is that is actually being measured.

Page 3 line 32 – the recent reference needs to be stated

Page 4 line 19. Explain more what "all possible" means

Page 5 line 21 … from reading the paper, the name "Boson Peak" would seem to have no useful meaning … can't some descriptive name be given that is intuitive and encapsulates what is going on? Perhaps the divided by frequency squared bit that comes later is important here ?

Page 51. line 31 needs references

Main reference:

Frohlich H (1968) Long-range coherence and energy storage in biological systems. Int J Quantum Chem 2:641–649.

Others:

Frohlich H (1968) Bose condensation of strongly excited longitudinal electric modes. Phys Lett A 26:402–403.

Frohlich H (1970) Long range coherence and the action of enzymes. Nature 228:1093. li

Page 6 top is the best explanation of what is being done. The quantity g(w)/w^2 is critical on page 13. No explanation is given as to why g is being divided by w^2 though that could help readers understand Fig 2. On page 6 at this point of the text, just saying alpha = g * w would help the readers much more. Also later (fig 1 etc) alpha is plotted in the figs not g, yet g is the quantity at the center of the discussion and the differences are marked as w ranges over a factor of 10.

At this point, the central assumption being used is that dmu/dr is independent of frequency, where mu is the dipole moment. Some justification for this key assumption should be given. Is the protein being considered as like one piece of elastic that just all expand and contracts together together? Or is the freeing of charged ions from captivity the central idea?

Page 10 bottom, and Fig 1b … the heat flow is presented as a negative number. How is it that this quantity can be negative (some quirk of the experiment) ? It is described in line 56 as decreasing … does this mean becoming enhanced ?

Page 11 lines 28 and 32 should be “dipole moment derivatives”

Page 13 top and previous lines … explain “jammed” and “confinement”

Page 14 lines 26-35 … it would help a lot if this explanatory text was copied into the introduction

Page 19 Frohlich condensate

I struggle with what is being said here. Frohlich’s model considers a system of oscillators at different frequencies that are anharmonically coupled and placed inside a thermal gradient in a steady state. It considers how the inherent non-equilibrium energy distribution manifests itself. The core prediction is that at high temperature a coherent condensate will form in which all of the internal system energy drives the mode of lowest frequency only.

I can envisage that this work can access all of the quantities involved as thermal energy flow is being measured in a non-equilibrium system, plus harmonic frequencies and anharmonic couplings. However, there is no attempt to map the experimental variables onto the parameters in the Frohlich model. Hence the work falls far short of its promise. The full numerical solution to the Frohlich model is available (www.pnas.org/cgi/doi/10.1073/pnas.0806273106) for one particular anharmonic-coupling model, so mapping the experiments onto that model would then allow Frohlic’s predictions to be manifested and tested. Most likely the experimental parameters are in a domain for which Frohlic’s model predicts no condensate. It would then be possible to determine how to modify the experiments so as to get an observable effect.

The lines in the text seem crazy too. Line 46 says that the absorption spectrums should sharpen as the temperature is lowered whereas in fact a critical aspect of the Frohlich model is that the absorption spectrum broadens and the condensate disappears at low temperature.

Line 54 claims that the Frohlich condensate is a quantum phenomenon. Indeed, Frohlich made such a claim. Frohlich used semiclassical statistical mechanics to derive his result, and hence the result could be quantum in nature. However, in the above PNAS reference, Frohlic’s equations are

rederived using classical mechanics. So there is no intrinsic connection with quantum effects. As line 56 etc state, dephasing will be very rapid in the systems studied and hence any observed coherence could only feasibly be classical in nature. A tornado is akin to a Frohlich condensate.

Reviewer: 2

Comments to the Author

1. What is the major advance reported in the paper?

Detection of the dynamic transition in anhydrous proteins by THz spectroscopy represents a major advance in understanding molecular mobility in proteins with very low water concentration. The dynamic transition has both theoretical significance (understanding protein function and role of water) and practical relevance (stability of pharmaceutical proteins).

2. What is the immediate significance of this advance?

Protein dynamic transition is mainly studied by neutron scattering, which is difficult to get access to. The study demonstrated that THz spectroscopy can be an orthogonal method to detect dynamic transition in proteins. In addition, this is a rare report of the dynamic transition in essentially anhydrous proteins. Insights into the nature of the dynamic transition, in particular that it does not have to involve solvent (water) molecules and that it involves the side groups (not protein backbone) are also significant.

From the practical perspectives of pharmaceutical stability, an important result is the detection of the PDT at approx. 230-240K for freeze-dried proteins, which is well below the glass transition temperatures reported for anhydrous proteins. For HSA, e.g., two Tg were reported at 325K and 350K (V. F. Uryash, N. Yu. Kokurina. Effect of Water on Physical Transitions of Human Serum Albumin. Polymer Science, Ser. A, 2011, 53, 1047–1053). Difference between HSA and lysozyme in the confinement (p. 13) is also of interest, at least for pharmaceutical scientists, as it may reflect difference in the “intrinsic” stability between these popular model proteins. Finally, relevance of the study to the Frolhlich condensate is very intriguing.

3. Technical suggestions

- It would be worthwhile to provide an estimate of residual water in the freeze-dried macromolecules. It is probably well below 1 wt%, based on the lyophilization protocol.

- p. 10, line 23: Please add reference to “It is known that sucrose…”

Reviewer: 3

Comments to the Author

Review of The Dynamical Transition in Dehydrated Proteins

Reviewer

February 10, 2024

General comments

This is an interesting paper in which THz spectroscopy is used to probe protein dynamics across a range of temperatures. The possibility of Fr¨olich coherences is investigated.

The work is done with care and thought and the experiments do shed some light on mobility in proteins. By using the absorbance of he samples at 1 THz it is possible to observe onset of mobility up to the so-called jamming regime (Figure 1). This is a very nice set of experiments. I note that the α_1 THz has a series of errors bars - are these from repeated measurements of the absorbance for differing replicate samples?

Specific suggestions

2.1 Page 6 , line 6

I do wonder if the statement "The excess density of states, apparent in the reduced density of states, is referred to as the boson peak (BP). The BP is a harmonic phenomenon due to inherent disorder that anharmonic effects can obscure." is all that informative. I realise the boson peak is difficult to explain but I think that saying excess density of states then reduced density of states is a bit misleading to someone who has not read a few boson peak papers. I suggest some rewording here.

Author's Response to Peer Review Comments:

Manuscript ID: jz-2023-03584s

Title: The Dynamical Transition in Dehydrated Proteins

We would like to thank the reviewers for their comments and suggestions. In order to facilitate the review process, we list all our responses and changes in this document point-by-point and have also incorporated the changes into the revised manuscript. All changes in the manuscript were highlighted in red in the revised version.

Reviewer #1:

This is an interesting paper in which THz spectroscopy is used to probe protein dynamics across a range of temperatures. The possibility of Frölich coherences is investigated.

The work is done with care and thought and the experiments do shed some light on mobility in proteins. By using the absorbance of the samples at 1 THz it is possible to observe onset of mobility up to the so-called jamming regime (Figure 1). This is a very nice set of experiments.

We would like to thank the reviewer for carefully reading our work and for the constructive comments provided.

1. I note that the α_1 THz has a series of errors bars - are these from repeated measurements of the absorbance for differing replicate samples?

This is a very good point. As stated in the caption of Figure 1, the error bars are the standard error for *n* measurements (*n*=5 for sucrose, *n*=4 for bacitracin, *n*=3 for lysozyme and HSA). Each measurement was performed on a different replicate sample.

2. Page 6, line 6: I do wonder if the statement "The excess density of states, apparent in the reduced density of states, is referred to as the boson peak (BP). The BP is a harmonic phenomenon due to inherent disorder that anharmonic effects can obscure." is all that informative. I realise the boson peak is difficult to explain but I think that saying excess density of states then reduced density of states is a bit misleading to someone who has not read a few boson peak papers. I suggest some rewording here.

Thank you for this comment. This wording might be misleading. We have changed the text as follows:

> The boson peak (BP) refers to an excess in the density of states with respect to Debye's $g(\omega) \propto \omega^2$ law, which can appear as a peak in the reduced density of states.

Reviewer #2:

Detection of the dynamic transition in anhydrous proteins by THz spectroscopy represents a major advance in understanding molecular mobility in proteins with very low water concentration. The dynamic transition has both theoretical significance (understanding protein function and role of water) and practical relevance (stability of pharmaceutical proteins).
Protein dynamic transition is mainly studied by neutron scattering, which is difficult to get access to. The study demonstrated that THz spectroscopy can be an orthogonal method to detect dynamic transition in proteins. In addition, this is a rare report of the dynamic transition in essentially anhydrous proteins. Insights into the nature of the dynamic transition, in particular that it does not have to involve solvent (water) molecules and that it involves the side groups (not protein backbone) are also significant.
From the practical perspectives of pharmaceutical stability, an important result is the detection of the PDT at approx. 230-240K for freeze-dried proteins, which is well below the glass transition temperatures reported for anhydrous proteins. For HSA, e.g., two Tg were reported at 325K and 350K (V. F. Uryash, N. Yu. Kokurina. Effect of Water on Physical Transitions of Human Serum Albumin. Polymer Science, Ser. A, 2011, 53, 1047–1053). Difference between HSA and lysozyme in the confinement (p. 13) is also of interest, at least for pharmaceutical scientists, as it may reflect difference in the "intrinsic" stability between these popular model proteins. Finally, relevance of the study to the Fröhlich condensate is very intriguing.

We would like to thank the reviewer very much for their comments and feedback.

1. It would be worthwhile to provide an estimate of residual water in the freeze-dried macromolecules. It is probably well below 1 wt%, based on the lyophilization protocol.

Thank you for pointing this out. Based on Karl-Fischer headspace analysis, the moisture content of all samples was at or below 0.2 wt.%. We have added the following to the manuscript:

> Karl-Fischer headspace analysis confirmed that the moisture content of all samples was at or below 0.2 wt.%.

2. p. 10, line 23: Please add reference to "It is known that sucrose…"

That is a good point. We added the following two references:
[31] Roe, K. D., and T. P. Labuza. "Glass transition and crystallization of amorphous trehalose-sucrose mixtures." International journal of food properties 8.3 (2005): 559-574.
[32] Kaminski, K., et al. "The true Johari− Goldstein β-relaxation of monosaccharides." The Journal of Physical Chemistry B 110.49 (2006): 25045-25049.

Reviewer #3:
I enjoyed reading this paper, which is suitable posed for JPC, and learnt a lot. So I recommend publication.

We would like to thank the reviewer for carefully reading our work and their constructive comments.

1. The language of this paper is often unfamiliar with phys chem teaching and practice. With this comes new phenomena that physical chemists have not focused on before. Since the 19th century such spectra have been recorded in wavenumbers not THz. The use of such a physically meaningful unit provides insight that can never be provided when SI units are used. This paper already uses wavenumbers whenever physical insight is evoked from the readers, but the main results are not presented in widely understood language. If I have done it right, then the frequency range used in the paper of 0.3 to 3 THz corresponds to 10-100 wavenumbers ... telling people this tells the audience what it is that is actually being measured.

Thank you for pointing this out. We have added the following to the introduction:

> […] between 0.3 THz and 3 THz (corresponding to 10 cm^{-1} to 100 cm^{-1}) […]

2. Page 3 line 32 – the recent reference needs to be stated

We would like to thank the reviewer for raising this point. The reference was originally placed at the end of the paragraph. We moved it further up now.

3. Page 4 line 19. Explain more what "all possible" means

We would like to thank the reviewer for this comment. We have amended the sentence to make it clearer:

> Instead of studying solvated biomolecules, we implemented an extensive lyophilisation to remove as many water molecules as possible from protein samples.

4. Page 5 line 21 ... from reading the paper, the name "Boson Peak" would seem to have no useful meaning ... can't some descriptive name be given that is intuitive and encapsulates what is going on? Perhaps the divided by frequency squared bit that comes later is important here ?

The name "boson peak" has historically been used to describe that phenomenon because its maximum intensity observed in scattering experiments at very low temperatures follows the frequency/temperature dependence of the Bose function.
These are some of the earliest papers:
P. Flubacher, A. J. Leadbetter, and J. A. Morris. J. Phys. Chem. Solids, 12:53, 1959.
A. J. Leadbetter and J. A. Morrison. Phys. Chem. Glasses, 4:188, 1963.
A. J. Leadbetter. Phys. Chem. Glasses, 9:1, 1968.
A. J. Leadbetter. J. Chem. Phys., 51:779, 1969.

R. Shuker and R. W. Gammon. Phys. Rev. Lett., 25:222, 1970.

5. Page 5. line 31 needs references Main reference:
   Frohlich H (1968) Long-range coherence and energy storage in biological systems. Int J Quantum Chem 2:641–649.
   Others:
   Frohlich H (1968) Bose condensation of strongly excited longitudinal electric modes. Phys Lett A 26:402–403.
   Frohlich H (1970) Long range coherence and the action of enzymes. Nature 228:1093. Li

We would like to thank the reviewer for pointing this out. We have added the suggested references to the text.

6. Page 6 top is the best explanation of what is being done. The quantity g(w)/w^2 is critical on page 13. No explanation is given as to why g is being divided by w^2 though that could help readers understand Fig 2. On page 6 at this point of the text, just saying alpha = g * w would help the readers much more.

Thank you for this comment. We have changed the text as follows to clarify this:

> The boson peak (BP) refers to an excess in the density of states with respect to Debye's $g(\omega) \propto \omega^2$ law, which can appear as a peak in the reduced density of states.

7. Also later (fig 1 etc) alpha is plotted in the figs not g, yet g is the quantity at the center of the discussion and the differences are marked as w ranges over a factor of 10.

We would like to thank the reviewer for raising this point. We decided to plot the α that we measured directly because it is firstly more familiar to readers and secondly because the errors at lower frequencies are reduced (we do not need to divide by a small number). But you are right, we could also have plotted α/ω.

8. At this point, the central assumption being used is that dmu/dr is independent of frequency, where mu is the dipole moment. Some justification for this key assumption should be given. Is the protein being considered as like one piece of elastic that just all expand and contracts together? Or is the freeing of charged ions from captivity the central idea?

We think of the proteins molecules as containing a large number of independent dipoles that can either couple individually with the terahertz field when they are located on sufficiently flexible parts of the molecules, i.e. only bound by low energy potentials, or that can couple collectively to the field if they are part of a more rigid structural motif of the biomolecule.

9. Page 10 bottom, and Fig 1b ... the heat flow is presented as a negative number. How is it that this quantity can be negative (some quirk of the experiment) ? It is described in line 56 as decreasing ... does this mean becoming enhanced ?

We would like to thank the reviewer for pointing this out. Because the heat flow is measured relative to a reference, it can be lower than 0. As indicated in the subfigure for sucrose, exothermic events are characterised by a relative increase in signal, and endothermic events therefore by a decrease.

10. Page 11 lines 28 and 32 should be "dipole moment derivatives"

We would like to thank the reviewer for this comment. The intensity of infrared active modes is proportional to the change in dipole moment μ upon displacement of molecules:

$$A_p \propto \left| \frac{\partial \mu}{\partial q_p} \right|^2$$

where A_p and q_p are the intensity and displacement vectors of the pth mode, respectively. In the text, we described it in terms of "changes" of the dipole moments, rather than derivatives. Both ways should be correct.

11. Page 13 top and previous lines ... explain "jammed" and "confinement"

We would like to thank the reviewer for pointing this out. We introduced the concept of jamming in the introduction but we extended the explanation on page 13 as follows:

> This phenomenon was previously observed in other (more complex) lyophilised formulations and attributed to high-temperature macromolecular confinement, strongly reducing the molecular mobility and resulting in a "jammed conformation". [27]

12. Page 14 lines 26-35 ... it would help a lot if this explanatory text was copied into the introduction

We would like to thank the reviewer for this suggestion. We have moved the paragraph to the introduction.

13. Page 19 Frohlich condensate
I struggle with what is being said here. Frohlich's model considers a system of oscillators at different frequencies that are anharmonically coupled and placed inside a thermal gradient in a steady state. It considers how the inherent non-equilibrium energy distribution manifests itself. The core prediction is that at high temperature a coherent condensate will form in which all of the internal system energy drives the mode of lowest frequency only.
I can envisage that this work can access all of the quantities involved as thermal energy flow is being measured in a non-equilibrium system, plus harmonic frequencies and anharmonic couplings.
However, there is no attempt to map the experimental variables onto the parameters in the Frohlich model. Hence the work falls far short of its promise. The full numerical solution to the Frohlich model is available (www.pnas.org/cgi/doi/10.1073/pnas.0806273106) for one particular anharmonic-coupling model, so mapping the experiments onto that model would then allow Frohlic's predictions to be manifested and tested. Most likely the

experimental parameters are in a domain for which Frohlic's model predicts no condensate. It would then be possible to determine how to modify the experiments so as to get an observable effect.

We would like to thank the reviewer for this comment. The paper mentioned gives a good theoretical overview and describes the occurrence of weak condensates as possible under biological conditions. There have been some reports of a possible condensate in the protein BSA (Phys Rev X. 8, 031061 (2018)) when optically pumped (a resonance was found at 0.3 THz, which lies within our frequency range). With our current set-up, we did not observe a coherent state. However, we added the following to the text:

> Our data hence show no evidence for Fröhlich coherence or the existence of a Fröhlich condensate or other quantum effects in a system that is not actively being pumped. [48]
>
> [48] Nardecchia, Ilaria, et al. "Out-of-equilibrium collective oscillation as phonon condensation in a model protein." Physical Review X 8.3 (2018): 031061.

14. The lines in the text seem crazy too. Line 46 says that the absorption spectrums should sharpen as the temperature is lowered whereas in fact a critical aspect of the Frohlich model is that the absorption spectrum broadens and the condensate disappears at low temperature.

We would like to thank the reviewer for raising this point. This sentence was meant to describe in general how a coherent mode appears in a terahertz spectrum. However, as has also been pointed out in the literature, (see for example Zhang et al., PRL 122.15 (2019): 158101), the emergence of a coherent state at a certain temperature would indeed result in a sharp feature in a terahertz (or Raman) spectrum.
We took out the part about the temperature dependence to avoid misunderstandings:

> "In terahertz spectra, such coherent modes appear as peaks in the absorption spectrum."

15. Line 54 claims that the Frohlich condensate is a quantum phenomenon. Indeed, Frohlich made such a claim. Frohlich used semiclassical statistical mechanics to derive his result, and hence the result could be quantum in nature. However, in the above PNAS reference, Frohlic's equations are rederived using classical mechanics. So there is no intrinsic connection with quantum effects. As line 56 etc state, dephasing will be very rapid in the systems studied and hence any observed coherence could only feasibly be classical in nature. A tornado is akin to a Frohlich condensate.

We would like to thank the reviewer for pointing this out. There are numerous studies in the literature with differing views as to whether Fröhlich coherence can be fully understood using only classical mechanics (see for example Zhang et al., PRL 122.15 (2019): 158101; Preto, J. Biol. Phys. 43.2 (2017): 167-184). As we did not perform any modelling, we decided to follow Fröhlich's terminology.
